# B7-H3 (CD276) as Target for T Cell-Based Bispecific Antibody Therapy of Penile Cancer

**DOI:** 10.2147/ITT.S577364

**Published:** 2026-05-01

**Authors:** Aleksander Kielbik, Veronika Bahlinger, Christian M Schürch, Maria Luisa Barcena, Olesya Vakhrusheva, Anita Thomas, Igor Tsaur, Jonas S Heitmann, Helmut R Salih, Ilona Hagelstein

**Affiliations:** 1 Clinical Collaboration Unit Translational Immunology, Department of Internal Medicine, University Hospital Tuebingen, Tuebingen, Germany; 2 Department of Urology, University Hospital Tuebingen, Tuebingen, Germany; 3 Cluster of Excellence iFIT (EXC 2180) “Image-Guided and Functionally Instructed Tumor Therapies”, University of Tuebingen, Tuebingen, Germany; 4 Department of Pathology and Neuropathology, University Hospital and Comprehensive Cancer Center Tuebingen, Tuebingen, Germany; 5 Department of Urology and Pediatric Urology, University Medical Center Mainz, Mainz, Germany; 6 German Cancer Consortium (DKTK), Partner Site Tuebingen, a Partnership Between DKFZ and University Hospital Tuebingen, Tuebingen, Germany

**Keywords:** penile cancer, bispecific antibody, immunotherapy, CD3, B7-H3, CD276, T cell

## Abstract

**Introduction:**

Metastatic or locally advanced penile cancer (PeCa) has limited systemic treatment options and a 5-year survival rate of ~10% in metastatic disease. Using the in vitro and ex vivo models we preclinically assessed CC-3, a B7-H3xCD3 bispecific antibody (bsAb) currently in a Phase I basket trial (NCT05999396).

**Methods:**

Primary PeCa cells were isolated from surgical specimens and characterized for surface antigen expression by flow cytometry (n = 4). B7-H3 expression was additionally evaluated in primary penile cancer tissues by immunohistochemistry (n = 10). The functional activity of the bsAb CC-3 was assessed in co-culture assays with PBMC, analyzing cytotoxicity, cytokine release, and T cell activation.

**Results:**

Immunohistochemistry of tumors from ten PeCa patients revealed strong and consistent B7-H3 (CD276) expression, with a mean H-score of 200, in tumor cells and tumor-associated vasculature, potentially enhancing T cell influx upon targeting. In vitro and ex vivo co-cultures of primary PeCa cells with peripheral blood mononuclear cells from healthy donors and PeCa patients showed that CC-3 robustly activated CD4⁺ and CD8⁺ T cells, as defined by upregulation of CD69 and CD25, with donor-dependent kinetics. CC-3 also induced potent tumor cell lysis and T cell proliferation across all patient-derived tumor samples, whereas the isotype control had no effect, confirming target-restricted activity.

**Discussion:**

These results demonstrate the strong immunostimulatory capacity of CC-3 and validate B7-H3 as a relevant target for T cell-based immunotherapy in PeCa. Our findings support the ongoing inclusion of PeCa patients in the clinical CC-3 trial and encourage further development of B7-H3-directed strategies for this rare, understudied malignancy.

## Introduction

Penile cancer (PeCa) is associated with a poor prognosis and limited treatment options, particularly in cases of locally advanced disease with high-risk features or metastatic stages.^1^ Currently, the overall 5-year survival rate for patients diagnosed with localized disease reaches 81%.^2^ However, only about 40% of men are diagnosed at this early stage,^2^ and many may have undetected micrometastatic disease, which can lead to a rapid progression of PeCa.^3^ In more advanced disease involving bilateral or pelvic lymph nodes (N2-N3), the 5-year survival rate drops to only 10–20%, and it is even below 10% when extranodal disease is detected.^4^,^5^

At present, the most frequently used treatment regimen for advanced stages is platinum-based chemotherapy.^6^ Studies on taxane-based neoadjuvant chemotherapy such as paclitaxel, ifosfamide, and cisplatin (TIP) or docetaxel, cisplatin, and fluorouracil (TPF), have shown an objective response rate of 40–50% and a pathological complete response in approximately 13% of patients.^5^ Currently, no effective second-line chemotherapy options are available. Guidelines of the European Association of Urology strongly recommend that patients with progressive disease should be offered the opportunity to enroll in clinical trials, including those experimental therapies in phase I or basket trials.^7^

Immune checkpoint inhibitors such as nivolumab and cemiplimab have demonstrated limited efficacy in PeCa, with reported response rates of 14–17% as monotherapy and up to approximately 50% when combined with chemotherapy.^8^,^9^ Despite these rather modest responses, PeCa^10^,^11^ represents an immune-infiltrated tumor entity, with significantly higher densities of CD3⁺, CD8⁺, and CD20⁺ lymphocytes in tumor tissue compared to adjacent non-neoplastic epithelium.^10^ The immune contexture diverges substantially according to HPV status. HPV-positive tumors display pronounced intraepithelial CD3⁺/CD8⁺ infiltration with strong Th1 polarization (T-bet⁺) accompanied by increased FoxP3⁺/CD25⁺ regulatory T cells, reflecting a simultaneously inflamed yet immunoregulated microenvironment.^11^ In contrast, HPV-negative tumors are less T cell inflamed but exhibit a more immunosuppressive phenotype characterized by higher PD-L1 expression and enrichment of M2-polarized CD163⁺ tumor-associated macrophages, where high intratumoral CD163⁺ density and low stromal CD8⁺ infiltration correlate with lymph node metastasis and worse survival.^12^ Beyond lymphocyte-macrophage interactions, a CD147–CXCL8–CD15⁺ neutrophil axis further shapes a tumor-promoting inflammatory niche associated with inferior outcome.^13^

Within this complex immune landscape, the Th1/IL-12/IFN-γ axis emerges as a central yet dysregulated regulatory pathway. Tumor tissues exhibit IFN-γ expression correlating with advanced TNM stage, and elevated IL-12 immuno-expression independently predicts poorer cancer-specific survival^14^ Moreover, IFN-γ induces IDO1 expression in PSCC and correlates with PD-1/PD-L1 and CTLA-4, supporting the concept of IFN-γ–driven adaptive immune resistance.^15^

Collectively, these findings indicate that PeCa often displays a Th1-inflamed - yet functionally - restrained tumor immune microenvironment.^16^ This coexistence of immune activation and immune suppression may limit the efficacy of conventional immune checkpoint blockade alone. Therefore, immunotherapeutic approaches capable of directly redirecting effector T cells toward tumor cells, independently of endogenous antigen presentation and pre-existing T cell priming, represent a rational and potentially more effective strategy in PeCa.

One such approach is the use of CD3-targeting bispecific antibodies (bsAbs), which simultaneously engage CD3 on T cells and tumor-associated antigens on cancer cells, thereby inducing a targeted immune response. BsAbs have shown encouraging clinical efficacy in the treatment of relapsed or refractory lymphoma and multiple myeloma.^17^,^18^ Recently the FDA also approved bsAbs for treatment of solid tumors, including tarlatamab for small cell lung cancer and tebentafusp for uveal melanoma.^19^,^20^

As the efficacy of bsAb therapy critically depends on the selective expression of tumor-associated target antigens on cancer cells, we performed a screening of primary PeCa cells to identify potential surface antigens suitable for therapeutic targeting. Among the screened antigens, B7-H3 (also known as CD276) was found to be consistently overexpressed in all examined primary PeCa cells as well as in histopathological samples from PeCa patients. Available data indicates that expression of B7-H3 in healthy tissues is limited.^21^ Recent studies have also implicated B7-H3 in immune evasion mechanisms, suggesting a potential role as an immune checkpoint molecule and a correlation with prognosis und therapeutic response.^22–25^ Accordingly, B7-H3 has emerged as a broadly expressed and immunologically relevant tumor antigen in numerous malignancies, including prostate, breast, and non-small cell lung cancer, where it is associated with poor clinical outcomes and resistance to immune-mediated clearance.^26^,^27^ In addition to its proposed immunosuppressive functions, B7-H3 has been implicated in promoting angiogenesis, epithelial-mesenchymal transition, and metastasis, which further supports its candidacy as a multi-faceted therapeutic target.^28^,^29^ The consistent overexpression of B7-H3 in PeCa, coupled with its functional relevance in tumor progression and immune escape, provides a compelling rationale for its therapeutic targeting.

Based on these observations, we selected the B7-H3×CD3 bsAb CC-3 for further investigation in PeCa. CC-3 incorporates the functionally superior B7-H3 binder 7C4 combined with a CD3low configuration, which demonstrated substantially reduced induction of IFN-γ and IL-2 compared with CD3high variants, while preserving potent cytotoxic activity.^30^

CC-3 has previously demonstrated in vitro and in vivo efficacy in various cancer entities.^30–32^ In the current study, we assessed the therapeutic potential of CC-3 for PeCa treatment. Functional assays confirmed T cell activation, memory T cell proliferation and robust cytotoxic activity across all tested primary PeCa cells. Our findings position CC-3 as a promising therapeutic candidate for the treatment of metastatic PeCa and support the meanwhile ongoing inclusion of PeCa patients in ongoing basket clinical trial (NCT05999396).^33^

## Methods

### Primary PeCa Cells

PeCa cells were retrieved from surgical specimens obtained during partial or radical penectomy, or pelvic or inguinal lymphadenectomy. Subsequent pathological analysis confirmed the initial diagnosis (for donor characteristics see Supplementary Table S1). The study was approved by the Ethics Committee 232/2024BO2, 452/2024BO2) and informed written consent of the patients was obtained. The tissue was then minced and collected by centrifugation (480 g, 10 min, ambient temperature (a.t).). The sediment was resuspended in buffer containing collagenase (3000 U/mL) and hyaluronidase (1000 U/mL), and incubated under moderate agitation (37°C, 30 min). The proteolytic degradation was continued by adding fresh collagenase for 30 min at 37°C. Debris was removed by a cell strainer (70 μm mesh) and the filtrate was sedimented by centrifugation (150 g, 7 min, a.t). Cells were seeded in cell culture 25T flasks (Sarstedt, Nümbrecht, Germany) and cultivated in EpiCM Medium (ScienCell, Carlsbad, CA) and Epithelial Cell Growth Supplement (ScienCell) for a minimum of 3 passages before being used in the study (details of all reagents used in the study are provided in Supplementary Table S2).

### Screening of Surface Membrane Marker Expression

To evaluate the expression of surface markers, cells were stained with antibodies against B7-H3 (CD276), CD44, CD47, Endoglin (CD105), EpCAM (CD326), HER2 (CD340), LAG-3 (CD223), MUC1 (CD227), Nectin-4, PD-L1 (CD274), PSMA, STEAP1, TIM-3 (CD366), and TROP2 (Details on the antibodies are provided in Supplementary Table S3). Antibodies that were not directly conjugated to fluorophores were subsequently labeled using PE-conjugated secondary antibodies and analyzed separately. Live/dead cell discrimination was performed using 7-AAD (BioLegend, San Diego, CA). Staining was carried out in 96-well plates following standard surface staining protocols. Data acquisition was performed using a FACS Canto II or FACS Fortessa flow cytometer (BD Biosciences, San Diego, CA), and data were analyzed with FlowJo software (version 10.10, BD Biosciences, Ashland, OR). Median fluorescence intensities (MFIs) were first normalized to the corresponding isotype control MFIs to account for background staining. The resulting fold-change values were then log_1__0_-transformed for heat map visualization The percentage of antigen-positive cells was determined using gating strategies based on fluorescence-minus-one (FMO) controls.

### Immunohistochemistry Evaluation

Freshly excised PeCa tissues (n = 10 primary tumor cases and 2 lymph node metastases, totaling 12 slides) were fixed in formalin and embedded in paraffin (FFPE). The tumor samples were obtained from the biobank of the Institute of General and Molecular Pathology and Pathological Anatomy with collection spanning the years 2019 to 2024 (Detailed donor characteristics are provided in Supplementary Table S4). Written informed consent was obtained from all tissue donors prior to sample collection. Immunohistochemical analysis of the sections was conducted on an automated VENTANA BenchMark ULTRA system (Roche) following the manufacturer’s protocol, utilizing the routinely applied B7-H3 antibody RBT-B7H3 (Medac/Bio SB, dilution 1:75). Hematoxylin and eosin (H&E) staining was performed on the PeCa sections to confirm the diagnosis.

B7-H3 expression was evaluated independently by two observers (one pathology resident [VG] and one board-certified pathologist [CMS]), both blinded to clinical data. Inter-rater agreement was assessed using the intraclass correlation coefficient (ICC), yielding a value of 0.90, indicating reproducibility of the H-score assessment.

Quantitative scoring was restricted to membranous staining of tumor cells; cytoplasmic staining, when present, was recorded qualitatively but not included in the score. Tumor areas were distinguished from non-tumor compartments using morphological criteria supported by matching H&E sections. Stromal staining was not scored and was not included in the H-score. Vascular B7-H3 expression was assessed separately: endothelial structures were identified by CD31 co-staining, and vascular staining was documented qualitatively but not incorporated into the tumor-cell H-score.

The B7-H3 staining intensity was scored using a standardized system: 0 = no expression, 1 = weak but detectable expression, 2 = moderate yet clearly positive expression, and 3 = strong expression. Additionally, the percentage of cells showing positive staining was evaluated through two independent assessments. H-scores were defined for each section by the following formula:

H-score = (0×% of cells with intensity 0) + (1×% of cells with intensity 1+) + (2×% of cells with intensity 2+) + (3×% of cells with intensity 3+).

### Peripheral Blood Mononuclear Cells (PBMC)

Peripheral blood mononuclear cells (PBMC) of healthy donors and PeCa patients were obtained after informed written consent and isolated by density gradient centrifugation (Biocoll; Biochrom, Berlin, Germany) (detailed donor characteristics for PeCa patients are provided in Supplementary Table S5) PBMC were viably frozen and stored in liquid nitrogen. PBMC were obtained from anonymized blood-donor buffy coats; donor metadata (including sex, age, and health status) were not available to the investigators.

### Production and Purification of Bispecific Antibodies (bsAbs)

The production of the bsAb targeting B7-H3 and CD3, referred to as CC-3, along with a non-targeting isotype control antibody (MOPCxCD3), has been described previously.^30^,^34^ In brief, both constructs were expressed in ExpiCHO cells (Gibco, Carlsbad, CA) and purified from culture supernatants via affinity chromatography using MabSelect columns (GE Healthcare, Munich, Germany). Analytical and preparative size-exclusion chromatography was performed with Superdex S200 Increase 10/300 GL and HiLoad 16/60 columns (GE Healthcare) to ensure structural integrity and purity. Endotoxin levels were verified to be below 0.5 EU/mL using the EndoZyme II kit (bioMérieux, Marcy-l’Étoile, France), following the manufacturer’s protocol.

MOPCxCD3 is a format-matched non-targeting control bsAb that is identical to CC-3 (same IgG-based scaffold/Fc and the same affinity-reduced anti-CD3 arm), but carries an irrelevant MOPC-21 binding domain instead of the B7-H3 binder, thereby controlling for CD3- and scaffold-dependent effects in the absence of tumor-antigen targeting.

### Flow Cytometry

To evaluate T cell activation, degranulation and proliferation, PBMC were stained with fluorochrome-conjugated monoclonal antibodies against surface markers described in Supplementary Table S3. 7-AAD (BioLegend) was used for live- and dead-cell discrimination. Measurements were performed using a FACS Canto II or FACS Fortessa (BD Biosciences) and data was analyzed using FlowJo software. Directly prior to FACS measurement the cells were stained using the standard protocol.

The sequential gating strategy used for all co-culture flow cytometry analyses is shown in Supplementary Figure S1 and includes exclusion of beads/debris, live-cell gating (7‑AAD−), singlet discrimination, separation of PBMC and tumor cells based on CellTrace Violet labeling, and downstream gating on CD4+ and CD8+ T cells for CD69/CD25 and memory-marker analyses. Representative event counts at key gates are provided in Supplementary Figure S1.

### Analysis of Cytokine Secretion

To determine bsAb-induced cytokine release, PBMC were cultured with PeCa cells (E:T ratio 5:1) in the presence or absence of CC-3 or control (1 nM each). After 24 h, supernatants were collected from co-cultures and centrifuged to remove cellular debris. LEGENDplex™ assays (BioLegend) for analysis of IL-2, IL-10, IFN-γ, and TNF in supernatants was used according to the manufacturer’s instructions. Briefly, samples were incubated with fluorescently barcoded capture beads, followed by detection antibodies and streptavidin-PE. Data were acquired on a BDFACSLyric flow cytometer and analyzed using LEGENDplex Data Analysis Software to quantify cytokine concentrations. LEGENDplex assay sensitivities (defined by the manufacturer as minimum detectable concentration (MDC) + 2×SD) in cell-culture medium were 1.4 ± 0.5 pg/mL (IL-2), 0.9 ± 0.4 pg/mL (IL-10), 0.9 ± 0.6 pg/mL (TNF), and 1.3 ± 1.1 pg/mL (IFN-γ).

### Cytotoxicity Assays

To assess target cell lysis by flow cytometry, tumor cells were labeled with 2.5 μM CellTrace™ Violet Cell Proliferation Kit (Thermo Fisher Scientific, Waltham, MA, USA) and co-cultured with PBMC at an E:T ratio of 5:1. Co-cultures were treated with either the experimental antibody CC-3 or an isotype control at concentrations ranging from 5 nM to 0.0016 nM, or were left untreated. Calibration beads (Sigma-Aldrich, St. Louis, MO, USA) were added to each sample to ensure standardized acquisition volumes and enable accurate quantification of viable target cells. Following incubation, dead cells were identified and excluded based on uptake of the membrane-impermeable dye 7-AAD. The remaining CellTrace-positive, 7-AAD–negative population, representing viable target cells, was quantified and compared to the untreated control condition.

To analyze cytotoxicity in real time, the xCELLigence Real-Time Cell Analysis (RTCA) system (Roche Applied Science, Penzberg, Germany) was used. This impedance-based platform measures electrical impedance across gold microelectrodes embedded in the bottom of E-plates, generating a unitless cell index that reflects cell adhesion, viability, and morphology; decreases in cell index indicate loss of adherent viable tumor cells and/or reduced proliferative capacity. PeCa cells were seeded at 10,000 cells per well in E-Plate 96 (ACEA/Agilent/Roche format) and allowed to adhere for 24 h. PBMCs were then added at an effector:target (E:T) ratio of 5:1 in the presence of 1 nM CC-3 or isotype control, and impedance was recorded every 15 min for 72 h. For analysis, cell index values were normalized to the time point of PBMC/antibody addition (baseline set to 1 at t_0_) to account for inter-well differences in initial attachment and growth.

### T Cell Proliferation Assays

To analyze the proliferation of T cell memory subsets, healthy donor PBMC were co-cultured with tumor cells at an E:T ratio of 5:1 in 96-well plates, in the presence or absence of 1 nM CC-3 antibody or isotype control. After 3 days of incubation, PBMC from each well were transferred to a new 96-well plate containing freshly seeded tumor cells and were again exposed to CC-3 or isotype control. Following additional three days of incubation, T cell subsets were analyzed by flow cytometry and characterized based on CD45RO and CD62L expression as follows: effector memory T cells (CD45RO⁻CD62L⁻), central memory T cells (CD45RO⁻CD62L⁺), naive T cells (CD45RO⁺CD62L⁺), and effector T cells (CD45RO⁺CD62L⁻).

### Study Period

Experiments on cocultures were performed between December 2024 and June 2025. Primary cell and tissue collection for histochemical evaluation spanned the years 2019 to 2024.

### Statistics

Cytokine release following CC-3 treatment was visualized as a heat map after Z-score normalization across all groups for each cytokine. For statistical analysis, cytokine levels were normalized to the untreated control to calculate fold changes in concentration. Statistical comparisons with the isotype control were performed using a nonparametric Mann–Whitney test, with the Holm-Šidák method applied to correct for multiple comparisons.

Dose–response data for T cell activation, tumor cell killing and proliferation were analyzed using nonlinear regression based on the Hill equation. Statistical comparisons between different concentrations of CC-3, isotype control, and untreated samples were conducted using one-way ANOVA followed by Tukey for multiple comparisons. To improve clarity, statistical significance markers were omitted from the main graphs; detailed statistical annotations are provided in the Supplementary Figures.

The comparison between the population of activated T cells between 24 and 72 hours of incubation with PeCa cells and PBMC was performed using a nonparametric Mann–Whitney test, with the Holm-Šidák method applied to correct for multiple comparisons.

For the visualization of T cell proliferation, the t-distributed stochastic neighbor embedding (t-SNE) analysis of T cell populations was performed using the FlowJo software. Flow cytometry data were pre-gated on live, singlet lymphocytes, and relevant T cell markers (eg., CD4, CD8, CD45RO, and CD62L).

Dimensionality reduction was performed in FlowJo using the native t-SNE platform with the default Opt-SNE learning configuration. The input populations consisted of gated CD4⁺ and CD8⁺ T lymphocytes for memory T cell analysis and gated PBMCs for comparisons between PeCa patients and healthy donors. For PBMC analyses, samples were randomly downsampled to 135,000 events prior to t-SNE computation to ensure comparable cell numbers across samples.

t-SNE was calculated on compensated and biexponentally transformed fluorescence intensities using the following markers: CD4, CD8, CD62L, and CD45RO for memory T cell analysis, and CD3, CD4, CD8, CD56, HLA-DR, CD1c, CD141, CD123, CD19, and CD14 for PBMC analysis. Forward and side scatter parameters, time, and viability dye channels were excluded. Default t-SNE settings were applied (perplexity = 30; maximum iterations = 1000; learning rate [eta] = 4200). The k-nearest neighbor algorithm was VP-tree and gradient descent optimization was performed using the Barnes–Hut approximation.

For the memory T cell analysis, t-SNE visualization was followed by conventional manual gating using FMO controls to define naïve and memory subsets (as described above). Subsets were overlaid on the t-SNE map using color-coded annotations.

For PBMC datasets, clustering was performed using the FlowSOM plugin in FlowJo. A self-organizing map was generated using a 10×10 grid (100 nodes) with Euclidean distance. Nodes were subsequently metaclustered into k = 10 clusters using hierarchical consensus clustering. Clusters were annotated based on median marker expression profiles and their frequencies were calculated as the proportion of cells within each identified cluster relative to the total T cell population. Frequencies were expressed as percentages and compared between healthy donors (n=8) and PeCa patients (n=9). Comparisons of cluster frequencies between healthy donors and PeCa patient T cells were performed using a nonparametric Mann–Whitney test. To account for multiple comparisons, the Holm-Šidák method was applied for p-value adjustment.

Biological replicates (n) correspond to independent donors. Each biological sample was analyzed in 2–3 technical replicates per experiment.

Statistical significance was determined using p-values, with *p* < 0.05 considered significant; significance levels are indicated as follows: *p* < 0.05 *p < 0.01 **p < 0.001 ***and *p* < 0.0001 ****. Statistical analysis was performed using GraphPad Prism (version 10.1.1; GraphPad Software, San Diego, CA).

## Results

### B7-H3 Is Overexpressed in PeCa

**Figure 1:**
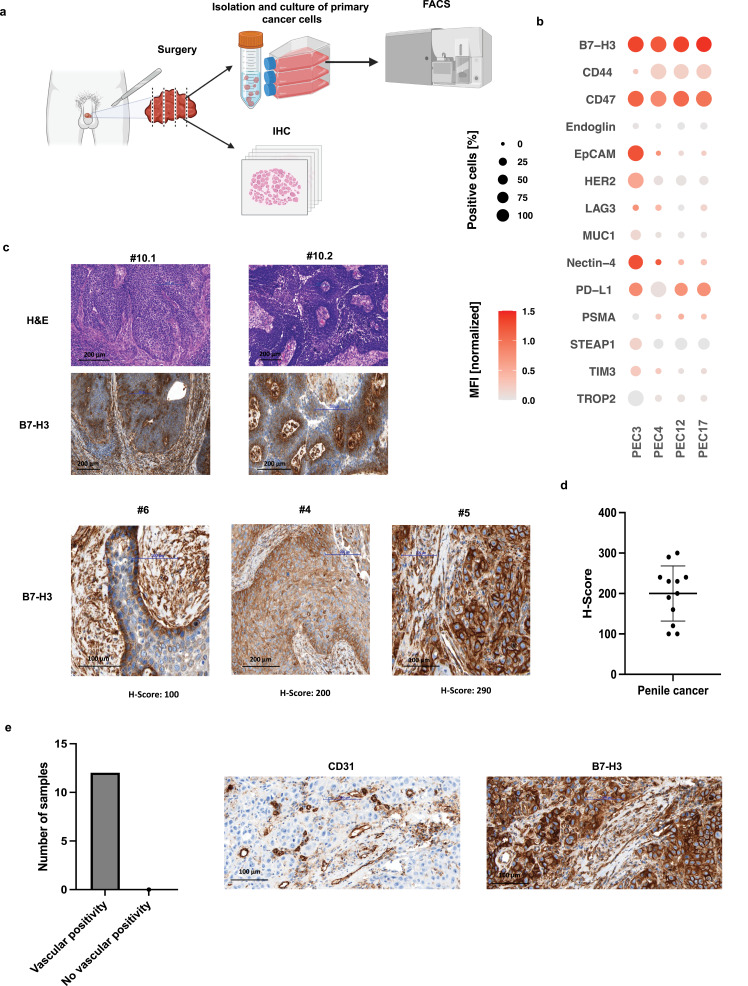
Characterization of B7-H3 expression in primary penile cancer (PeCa) cells and tissue, including tumor-associated vasculature. (**a**) Following penectomy or lymphadenectomy, primary PeCa cells were isolated and cultured in differentiation medium. The cells were then characterized using flow cytometry (FACS). Portions of tumor tissue were fixed, paraffin-embedded, and processed for histopathological evaluation. (**b**) Bubble heatmap showing the percentage of B7-H3–positive cells and corresponding median fluorescence intensities (MFIs). MFIs were first normalized to the isotype control to account for background staining, and the resulting fold-change values were log_1__0_-transformed for visualization. (**c**) Representative histological images of tumor tissue sections stained with hematoxylin and eosin (H&E) and immunohistochemistry for B7-H3 (20× magnification). Corresponding representative H-scores for B7-H3 expression are shown at 40× magnification. Samples #10.1 and #10.2 represent the primary tumor and the matched lymph node metastasis from the same patient, respectively. Samples #6, #4, and #5 show representative primary tumor images with different H-scores. Histopathological details of all specimens are provided in Supplementary Table S4. (**d**) Scatter plot summarizing B7-H3 expression levels in tumor tissues from n = 12 (n = 10 primary tumor cases and 2 lymph node metastases). (**e**) Bar plot showing that all analyzed tumors exhibited B7-H3 expression in vessels within the tumor microenvironment. Representative immunohistochemical images showing CD31 (endothelial marker) and B7-H3 staining in tumor sections (40× magnification).

Being a very rare urogenital cancer, the expression profile of potential therapeutic targets in PeCa has not yet been well established. Previous studies have identified PD-L1,^35^ HER2^36^ EGFR,^37^ TROP-2,^38^ nectin-4^39^ as possible targets; however, none of these have yet been explored as candidates for T cell-based therapy for PeCa. We performed a screening of patient-derived PeCa tissues for B7-H3 expression and primary PeCa cells for expression of tumor-associated antigens and immunoregulatory molecules that could serve as targets for bsAb treatment of PeCa (Figure 1a and Supplementary Table S1).

As a first step, we performed flow cytometry (FACS) analyses on primary PeCa cells (PEC3, PEC4, PEC12, PEC17) to assess the surface expression of the following antigens: B7-H3 (CD276), CD44, CD47, Endoglin (CD105), EpCAM (CD326), HER2 (CD340), LAG-3 (CD223), MUC1 (CD227), Nectin-4, PD-L1 (CD274), PSMA, STEAP1, TIM-3 (CD366), and TROP2. (Details on the antibodies are provided in Supplementary Table S3). Shown are combined results regarding both, the MFI and the percentage of positive cells across the panel of investigated surface antigens. The tested cells demonstrated substantial variability in the expression profiles of surface antigens. Among the analyzed antigens, B7-H3 showed expression in over 90% of cells, with a consistently high MFI across the four primary cultures. CD47, CD44, and PD-L1 were also found to be overexpressed in PeCa cells; however, their expression levels varied between samples, indicating a less consistent expression pattern (Figure 1b). These results highlight the potential of B7-H3 as a target for T cell therapy.

Next, we conducted histopathological analysis of specimens from 10 PeCa patients, including 10 primary tumors and 2 lymph node metastases. The patient cohort included individuals with varying TNM stages, histologic subtypes, and tumor grades (Supplementary Table S4). Corresponding to the FACS analysis, all specimens demonstrated substantial membranous B7-H3 staining with at least a minimum staining intensity of 2+ (moderate but clearly positive) in 20% of evaluated cells. In some cases, expression of B7-H3 was more pronounced at the basal compared to luminal layers (Figure 1c). None of the analyzed cases were negative for B7-H3 and the mean H-Score across all analyzed samples was 200 (Figure 1d). The expression of B7-H3 on tumor cells, with all specimens showing at least a subset of cells with high B7-H3 expression scores (2+ or 3+), indicates a pronounced positivity of B7-H3 in PeCa.

Additionally, B7-H3 expression was detected in tumor-associated vasculature across all analyzed cases. Co-staining with CD31, a marker of vascular endothelium, revealed a strong co-localization with B7-H3 in all examined PeCa samples (Figure 1e). The presence of tumor-associated target antigens in the tumor neovasculature is an important prerequisite for efficacy of bsAb therapies,^40–42^ further supporting the potential of B7-H3 as target for T cell-based immunotherapy of PeCa.

### CC-3 (B7-H3xCD3) Bispecific Antibody Induces Activation of T Cells Against PeCa

**Figure 2:**
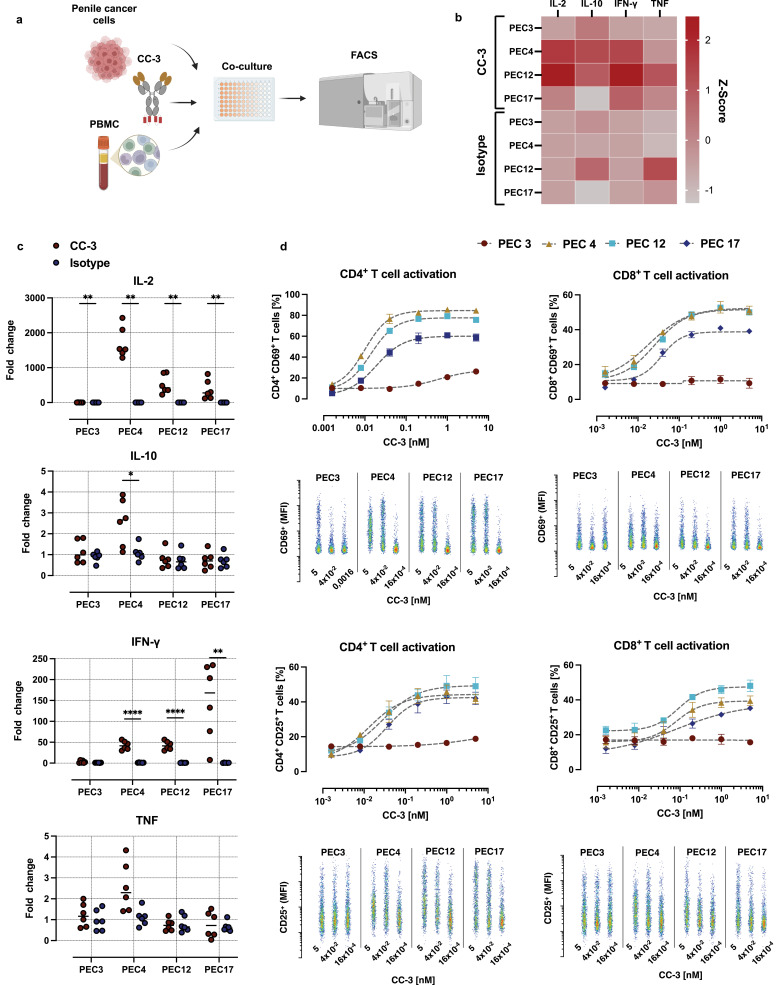
CC-3–mediated cytokine release and T cell activation in co-cultures of PBMC and primary PeCa cells. (**a**) Peripheral blood mononuclear cells (PBMC) were co-incubated with primary PeCa cells at an effector-to-target (E:T) ratio of 5:1 in the presence or absence of the bispecific antibody CC-3 or an isotype control. After incubation, cytokine release (IL-2, IL-10, IFN-γ, and TNF) and T cell activation were assessed by measuring surface expression of CD69 and CD25 on CD4⁺ and CD8⁺ T cells using flow cytometry. (**b)** Z-score–normalized cytokine concentrations measured 24 hours after exposure to CC-3 or the isotype control. Data represent the mean Z-scores derived from PBMC of three independent donors (n=3). (**c**) Fold-change in cytokine levels relative to the untreated control group. Shown are combined results of PBMC from three independent donors (n=3) with each data point corresponding to a single technical replicate. Statistical comparisons between CC-3 and isotype control groups were performed nonparametric Mann–Whitney test, with the Holm–Šidák method applied to correct for multiple comparisons. *p < 0.05; **p < 0.01; ****p < 0.0001. (**d**) CD4⁺ and CD8⁺ T cell activation, determined by flow cytometric analysis of CD69 and CD25 expression after 72 h. Results are pooled from three independent donors and are presented as mean ± SEM. For dose–response analysis of CC-3–mediated T cell activation data were fitted using nonlinear regression based on the Hill equation. Representative scatter plots show the MFI of activation markers as a function of CC-3 concentration.

Next, we employed our bsAb CC-3, which simultaneously targets B7-H3 and CD3^30^ in experimental settings of healthy donor PBMC and primary PeCa cells and analyzed cytokine release and T cell activation by FACS (Figure 2a). As first step, we assessed the release of key immunoregulatory cytokines within the first 24 hours following CC-3 application (Figure 2a–c). Analysis of culture supernatants using LEGENDplex assays revealed a significant increase in IL-2 and IFN-γ concentrations upon treatment with CC-3 (Figure 2b and Supplementary Figure S2), indicating that CC-3 effectively induces immune activation. The magnitude of this response varied across different primary cancer cell samples, underscoring the influence of tumor-specific factors on cytokine release. All tested cells showed a significant elevation in IL-2 release (p < 0.05), with the weakest response observed with PEC3 and the strongest with PEC4. Additionally, IFN-γ secretion was significantly increased using PEC4, PEC12, and PEC17 (p ≤ 0.01) and significant increase in IL-10 was detectable only in PEC4 cells (p< 0.05), and induction of TNF did not reach statistical significance in any of the tested samples (p > 0.05) (Figure 2c).

In subsequent experiments, CC-3 was added at various concentrations ranging from 0.0016 to 5 nM. T cell stimulation was assessed by measuring surface expression of CD69 and CD25, established markers of early and sustained T cell activation.^43^ After 24 hours of co-culture, variable degrees of T cell activation were observed among the different primary PeCa cell samples that corresponded with the induction of cytokines. PBMC incubated with PEC4, PEC12, and PEC17 PeCa cells exhibited robust expression of the activation markers CD25 and CD69 in both CD4⁺ and CD8⁺ T cell subsets, with activation reaching 30% or more of the respective populations. In co-cultures with these primary cells, significant activation of CD4⁺ T cells compared to the isotype control was observed at CC-3 concentrations as low as 0.04 nM (p < 0.01). For CD8⁺ T cells, CD25 expression became significantly elevated at 0.2 nM CC-3 (p < 0.01), whereas CD69 upregulation was detectable already at 0.04 nM (p < 0.001) (Supplementary Figure S3). Notably, no activation of CD8⁺ T cells was observed in PBMC co-cultured with PEC3 primary PeCa cells, even at the highest tested CC-3 concentration of 5 nM. CD4⁺ T cells showed modest but statistically significant activation reaching 20% of population, and thus markedly lower than the levels observed with the other primary cells PEC4, PEC12, and PEC17 (Figure 2d).

**Figure 3:**
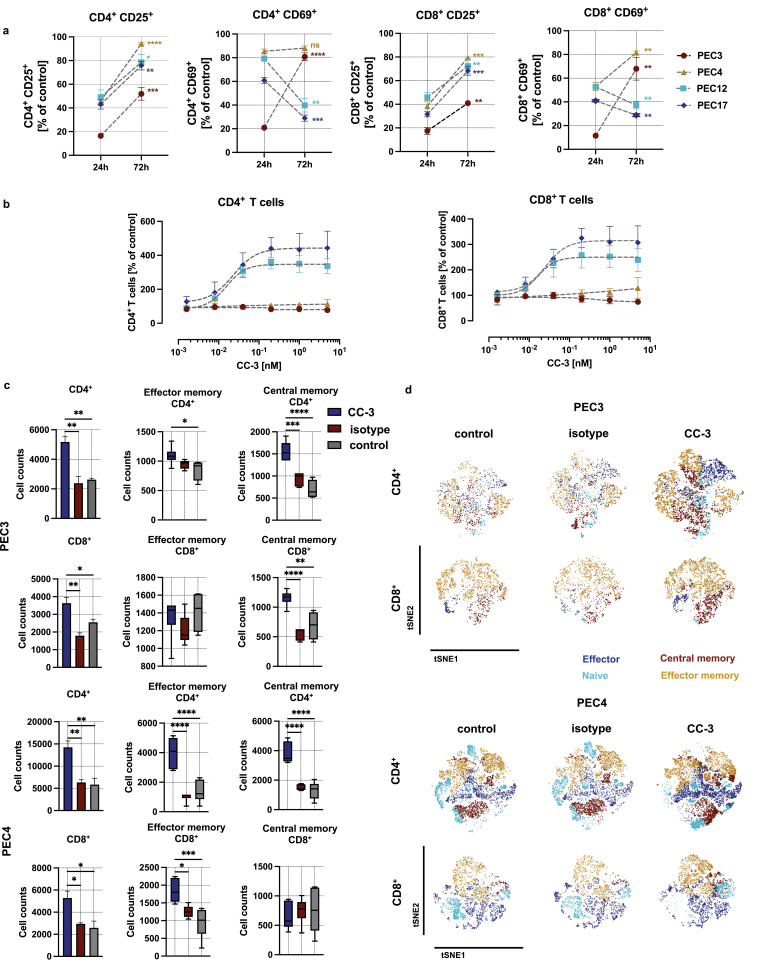
Long time activation and the induction of T cell proliferation and memory T cell populations by CC-3. PBMC were co-incubated with primary PeCa cells at an effector-to-target (E:T) ratio of 5:1 in the presence or absence of the bispecific antibody CC-3 or an isotype control. (**a**) Comparison of the percentage of activated T cells at 24h and 72 h following the incubation with CC-3. Data represent PBMC from three independent donors (n=3, mean ± SEM). The comparison of T cell activation between 24 and 72 h has been performed using nonparametric Mann–Whitney test, with the Holm–Šidák method applied to correct for multiple comparisons. (**b**) The dose response of CC-3 (T cell proliferation) was assessed by gating the CD4^+^ and CD8^+^ T cells after 72 hour incubation. Data were fitted using nonlinear regression based on the Hill equation. Data represent results obtained with PBMC from three independent donors (n=3, mean ± SEM). (**c**) Comparison of memory T cell counts 120 h after exposure of PEC3 and PEC4 to cells to PBMC in the presence of CC-3 or isotype control. After 120 h of incubation, CD4⁺ and CD8⁺ T cell subpopulations were analyzed by flow cytometry. T cell subsets were defined based on CD62L and CD45RO expression: CD62L⁻CD45RO⁻ cells were classified as effector T cells, CD62L⁺CD45RO⁻ as naïve T cells, CD62L⁻CD45RO⁺ as effector memory T cells, and CD62L⁺CD45RO⁺ as central memory T cells. Data of CD4^+^ and CD8^+^ T cells were presented as mean ± SEM (n=3 three independent donors). The count of the memory T cells is presented as a mean with error bars representing the min and max values, n=3. Statistical analysis of the T cell proliferation has been performed using the one-way ANOVA with Dunnett correction for multiple comparisons between CC-3, isotype and control groups. (**d**) The dimensionality reduction was conducted using the t-SNE algorithm with default parameters (perplexity, learning rate, and iterations), enabling visualization of proliferative dynamics within the T cell compartment. Statistical significance (panels a,c): *p < 0.05; **p < 0.01; ***p < 0.001; ****p < 0.0001.

After 72 h of exposure to CC-3, distinct T cell-activation patterns were observed depending on both the activation marker and the co-cultured cancer cells. CD25 expression was significantly increased in CD4⁺ and CD8⁺ T cells across all tested cells (p < 0.05), whereas CD69 expression in these T cell subsets varied depending on the co-cultured primary cell, significantly increasing with PEC3 and PEC4, but decreasing with PEC12 and PEC17 (p < 0.05) (Figure 3a and Supplementary Figure S4).

### CC-3 Induces T Cell Proliferation in a Tumor-Specific Manner

Induction of T cell proliferation is essential for therapeutic success, particularly to combat high tumor burden. To assess the influence of CC-3 treatment on T cell proliferation, PBMC were cultured for 72 hours with PeCa cells and treated with CC-3. Using two primary cells, PEC12 and PEC17, we observed a significant, dose-dependent increase in the number of CD4⁺ and CD8⁺ T cells, reaching a 3- to 4-fold expansion at CC-3 concentrations ranging from 0.04 to 5 nM (p < 0.01). T cells co-cultured with PEC3 and PEC4 cells, although activated, did not display detectable proliferation at CC-3 concentrations up to 5 nM after 72 h of incubation (p<0,05 for all concentrations) (Figure 3b and Supplementary Figure S5). This heterogeneous response prompted further investigations of proliferation at later time points, including a rechallenge assay in which PBMC were re-exposed to fresh PEC3 or PEC4 cells after the initial treatment. After six days of exposure, in the long-term co-cultures with PEC3 and PEC4 cells we observed a significant increase in both CD4⁺ and CD8⁺ memory T cell populations (p < 0.001 for CD4⁺; p < 0.05 for CD8⁺ with both PEC3 and PEC4 cells comparing CC-3 treated to control), showing a robust and sustained immunostimulatory response. Further analysis of the memory T cell subpopulations revealed that CD4⁺ effector and memory T cells were significantly elevated compared to both isotype-treated and untreated control conditions (p < 0.05 for both cells). Similarly, within the CD8⁺ compartment, effector T cell populations showed a significant increase (p < 0.05 CC-3 vs. control for both cells) (Figure 3c). To further assess the impact of CC-3 on T cell subset composition, t-SNE analyses were performed on PBMC co-cultured with either PEC3 or PEC4 primary PeCa cells. The longer incubation time resulted in a marked increase in effector and effector memory T cell populations within both CD4⁺ and CD8⁺ compartments. This shift was particularly pronounced in the CD8⁺ T cell population, where naive cells were replaced by effector and effector memory subsets upon CC-3 exposure. These findings suggest that CC-3 promotes T cell differentiation and functional polarization in a tumor-specific context, with a robust induction of effector phenotypes in all tested cells (Figure 3d).

### CC-3 Induces Dose-Dependent Cytotoxicity Against All Tested Primary PeCa Cells

**Figure 4:**
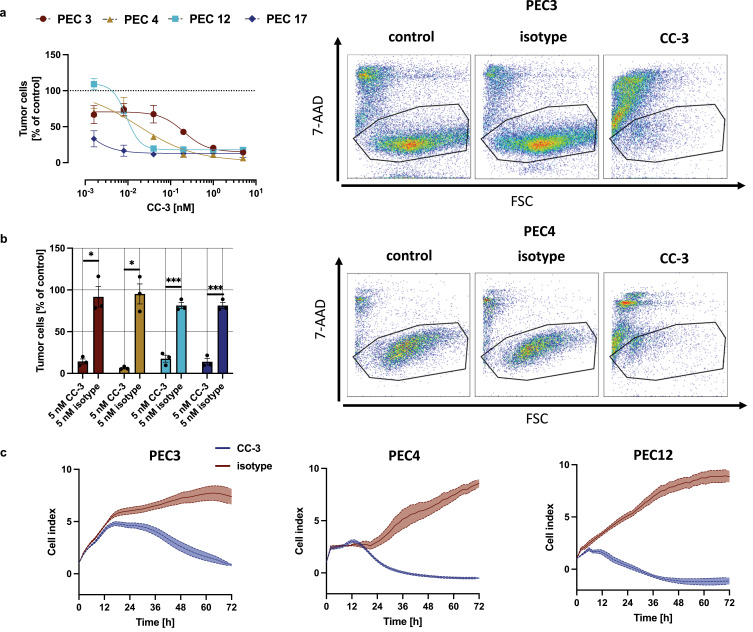
Dose-dependent, T cell mediated cytotoxicity of CC-3. (**a**) Cell lysis of primary penile cells was determined using a flow cytometry-based assay after 72 h. The right panels show exemplary scatter plots, while the left panels display combined dose-response data obtained from 3 independent donors of PBMC (n=3). The data are presented as mean ± SEM and were fitted using nonlinear regression based on the Hill equation. (**b**) PBMC were incubated with the indicated tumor cells (E:T 5:1) in the presence or absence of CC-3 or isotype control. To evaluate T cell–mediated tumor cell killing, the number of viable tumor cells (after exclusion of dead cells) was calculated as % of to the untreated control. The statistical comparison was performed using nonparametric Mann–Whitney test and Holm–Šidák method for multiple comparisons. *p < 0.05; ***p < 0.001. (**c**) The timeline of cytotoxic effects of PBMC from 3 independent donors (n=3) against PeCa were determined using the xCELLigence system. The data are presented as mean ± SEM.

Next, we analyzed how CC-3 affected PeCa cell viability after 72 hours of co-culture with PBMC from healthy donors. Cancer cell death was assessed using flow cytometry for 7-AAD, a well-established marker of compromised membrane integrity. With all tested PeCa cells, a dose-dependent decrease in the number of viable cancer cells was observed. The calculated average dose of CC-3 which kill 50% of cells (LD_5__0_) varied with different cells, ranging from as low as 0.0006 nM with the PEC17 to 0.2 nM with PEC3 cells (Figure 4a). At a CC-3 concentration of 5 nM, pronounced cytotoxicity was observed in all tested co-cultures with primary PeCa cells, achieving almost complete tumor cell elimination (<15% of viability. p < 0.05 for all primary cell cultures), whereas the isotype control did not induce measurable cytotoxic effects (Figure 4b). To quantify the dynamics of the antitumor effect, tumor cells were co-cultured with PBMC and CC-3 and monitored real-time for cell adhesion. A decrease in cell index, indicating reduced cell adhesion and viability, was observed between 6 hours and 18 hours following CC-3 exposure in co-culture, shedding light on the onset and progression of cytotoxic activity. (Figure 4c). In summary, CC-3 potently induced tumor cell killing in all investigated primary PeCa cells.

### CC-3 Successfully Activates T Cells From PeCa Patients, Triggering a Robust Cytotoxic Response

PBMC from cancer patients often are less responsive to immunotherapeutic stimulation compared to those from healthy donors. This diminished functionality may be attributable to T cell exhaustion and impaired cytokine production and accompanied by an increase in regulatory T cells and myeloid-derived suppressor cells, which serve to dampen immune activation.^44–46^

**Figure 5:**
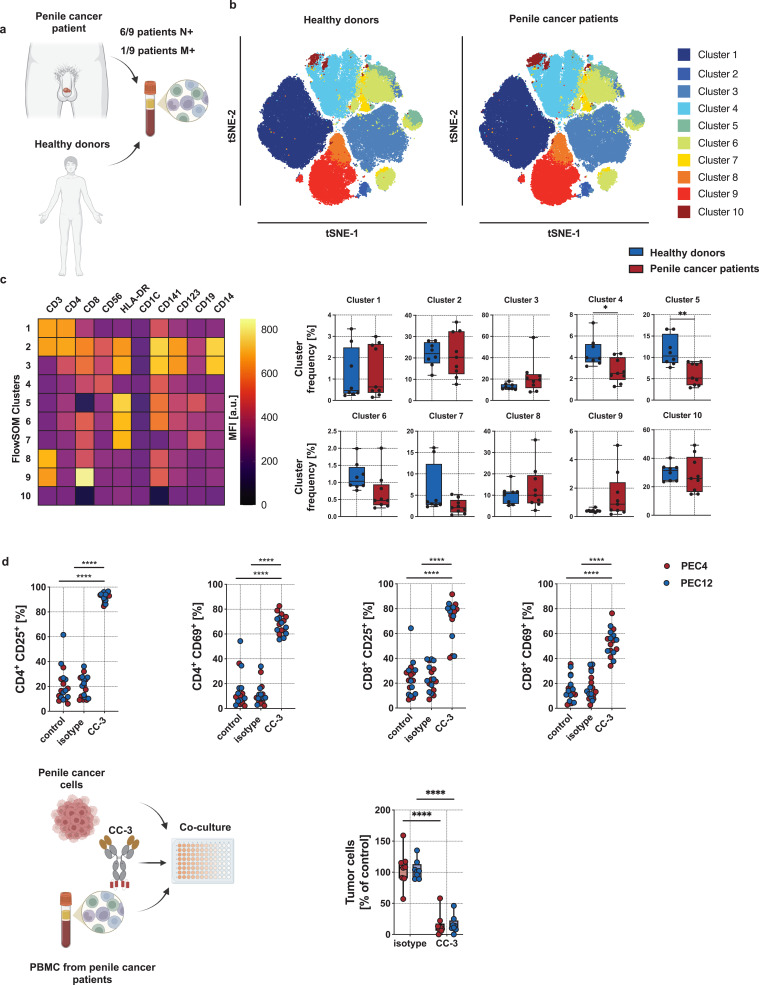
Phenotyping of healthy donor and PeCa patient PBMC and ex vivo analyses of CC-3–mediated T cell reactivity against PeCa with PBMC from PeCa patients. (**a**) Blood samples from eight healthy donors and nine PeCa patients were collected followed by isolation of PBMC. (**b**) Phenotyping of PBMC was performed using flow cytometry. The dimensionality reduction was conducted using the t-SNE algorithm with default parameters (perplexity, learning rate, and iterations), enabling clustering of subsets within PBMC. (**c**) For cluster description, the expression of lymphocyte markers within each FlowSOM cluster is shown as MFI. (left panel). Right panels compere cells frequency in each cluster between healthy donor (n = 8) and PeCa patient (n = 9). Comparisons of cluster frequencies between healthy donors and PeCa patient T cells were performed using a nonparametric Mann–Whitney test with Holm–Šidák method for p-value adjustment. (**d**) PeCa patient PBMC were incubated with the indicated tumor cells (E:T 5:1) in the presence or absence of CC-3 or isotype control (n = 9). T cell activation was analyzed after 72 h of incubation by expression of CD25 and CD69 by flow cytometry (upper panels). To evaluate T cell–mediated tumor cell killing, the number of viable tumor cells (after exclusion of dead cells) was calculated as % of to the untreated control (lower panels). Statistical significance (panels c-d): *p < 0.05; **p < 0.01; ****p < 0.0001.

To analyze, if these potential differences in effector cells within PBMC influence the therapeutic potential of CC-3, we performed comparative analyses using PBMC of healthy donors and PeCa patients. Among nine PeCa patients who donated PBMC, six had presented with lymph node metastases and one had confirmed distant metastasis. None of the patients had received systemic therapy prior to PBMC collection (Figure 5a and Supplementary Table S5).

The comparison of subpopulations and clusters from healthy donors and PeCa patients revealed a significant difference in the percentage of cells phenotypically corresponding to dendritic cells. Aside from this, no other changes in immune cell composition were observed (Figure 5b, c and Supplementary Figure S6).

Analysis of T cell activation demonstrated that patient PBMC co-cultured with PEC4 and PEC12 cells exhibited robust activation following 72 hours of incubation with 1 nM CC-3. This activation was evidenced by a significant increase in CD69 and CD25 expression on both CD8⁺ and CD4⁺ T cells compared to isotype and untreated controls (p < 0.0001; Figure 5d). The proportion of T cells expressing activation markers was comparable to that observed with PBMC from healthy donors, indicating preserved T cell responsiveness in cancer patients upon treatment with CC-3. Moreover, 72-hour incubation with 1 nM CC-3, induced a potent cytotoxic response of PBMC from PeCa patients against PeCa cells (p < 0.0001 vs. isotype control) (Figure 5d).

## Discussion

BsAbs represent a rapidly advancing class of immunotherapies with the capacity to redirect T cells toward tumor cells through CD3 engagement. Several other CD3-engaging bsAbs targeting prostate-specific membrane antigen (PSMA), human epidermal growth factor receptor 2 (HER2), mucin-1 (MUC1), and six-transmembrane epithelial antigen of the prostate 1 (STEAP1) are currently under clinical investigation.^34^,^47^ We have developed CC-1, a PSMA×CD3 bispecific antibody, that presently is being evaluated in metastatic prostate cancer (mCRPC) (NCT04104607)^48^ as well as biochemical relapse of prostate cancer. A STEAP1×CD3 bsAbs developed by AMGEN (AMG 509) is under investigation in prostate cancer (NCT04221542). MUC1×CD3 bsAbs such as SAR564844 are in early-phase studies for non-small cell lung cancer and pancreatic cancer (NCT05239143)). HER2-targeted CD3 bsAbs, including PRS-343, are being studied in HER2-positive solid tumors (NCT03650348). Furthermore, trophoblast cell surface antigen 2 (Trop-2), a clinically validated target for antibody-drug conjugates, is gaining attention as a candidate for future CD3-engaging bsAb development in epithelial tumors due to its high expression across a range of epithelial tumors.^49^ To date, no bsAbs, including those that are approved for other solid tumors, are being evaluated for treatment of PeCa, underscoring a critical gap in therapeutic development for this rare disease.

To date, B7-H3 expression has not been characterized in PeCa. Our findings show uniform overexpression of B7-H3 in all patient samples analyzed. Comprehensive immunohistochemical profiling across a broad spectrum of human malignancies have reported strong B7-H3 expression with minimal staining in most normal tissues.^50^ Nonetheless, low-level expression has been described in selected non-malignant compartments, including antigen-presenting cells, endothelial cells, and fibroblasts, and both preclinical and early clinical data support the therapeutic tractability of B7-H3 in solid tumors.^28^ Taken together, these findings justify continued investigation while underscoring the importance of dedicated cross-reactivity and safety assessment of CC-3 in future studies.

Clinical trials investigating B7-H3-directed antibody-drug conjugates (ADCs), including vobramitamab duocarmazine, YL201, and HS-20093, have shown encouraging antitumor activity, albeit with some toxicity likely attributable to the cytotoxic payloads.^51–53^ Notably, agents like GSK5764227 have received FDA Breakthrough Therapy Designation, further supporting B7-H3’s clinical relevance. In addition, emerging strategies such as B7-H3-targeting CAR T cells and bsAbs have demonstrated potent anti-tumor effects in preclinical models, reinforcing the feasibility of B7-H3-directed immunotherapy.^54–56^

In this study, the in-vitro and ex-vivo data confirm that B7-H3×CD3 bsAb CC-3 effectively induced T cell antitumor immunity against in primary PeCa cells when co-cultured with PBMC from both healthy donors and PeCa patients. T cell activation was confirmed by increased surface expression of CD69 and CD25 on both CD4⁺ and CD8⁺ T cells. This activation correlated with significant cytotoxic effects and T cell proliferation across all tested primary PeCa cells. Despite uniform B7-H3 overexpression on the surface of cancer cells, subtle yet consistent variability in immune responses was observed across different primary PeCa cells following exposure to CC-3. These differences encompassed the magnitude and timing of cytokine release, the kinetics of T cell activation, and the extent of memory T cell proliferation. Such tumor-specific variations may reflect downstream resistance mechanisms, including impaired co-stimulatory signaling or disrupted immune synapse formation in PeCa cells.^57^,^58^

In our study, higher cytokine levels were observed in parallel with increased cytotoxicity and T cell activation after CC-3 treatment. However, previous analyses of the penile cancer microenvironment have shown that elevated immunostimulatory cytokine expression may also be associated with advanced tumor stage.^14^,^15^ Consequently, cytokine elevation alone cannot be considered a reliable surrogate marker for therapeutic success and should be interpreted within the broader functional and clinical context.

Nevertheless, the high and consistent cytotoxic effect highlights the capacity of CC-3 to redirect T cells for direct target cell killing via CD3 engagement, even in the absence of full T cell activation or cytokine release, as has already been observed in the context of other T cell-targeting therapeutic strategies.^59^,^60^ Our study is among the first to utilize primary PeCa tumor cells and patient-derived PBMC in functional immunological assays. By capturing the biological heterogeneity of penile tumors ex vivo, our data offer a clinically relevant demonstration of the potential of CC-3 to overcome tumor immune resistance in patient-specific contexts.

Importantly, CC-3 also induced strong T cell activation and cytotoxicity with PBMC derived from PeCa patients, comparable to responses observed with PBMC from healthy donors. Despite the immunosuppressive environment typically associated with cancer, including T cell exhaustion and expansion of suppressive immune populations, PeCa patient derived PBMC retained responsiveness to CC-3. Aside from a reduced frequency of dendritic cells, immune subset composition was largely preserved. This underscores the feasibility of T cell redirection via bsAbs even within the context of cancer where immunological function may be compromised.

Beyond tumor cell expression, we also assessed the endothelial localization of B7-H3 through co-staining with the vascular marker CD31. Consistent with previous findings [10], CD276 was strongly expressed in tumor-associated vasculature in 51% of tumors examined, whereas strong staining of tumor cells was observed in 30% of cases. In contrast, staining in normal tissues was largely absent. Only low-level reactivity was detected in sinusoidal endothelial cells of the liver and in epithelial cells of the skin, bladder, uterus, and esophagus. Notably, this weak signal may partially reflect non-specific cross-reactivity of the polyclonal antibody used in that study. The presence of B7-H3 in tumor-associated vasculature may have important therapeutic implications, particularly for CD3-engaging formats. Targeting tumor endothelial cells could theoretically augment antitumor efficacy by inducing selective disruption of tumor vessels, promoting vascular collapse, and facilitating immune cell infiltration into the tumor microenvironment.^61^

However, detectable expression on normal endothelial cells necessitates careful consideration of potential on-target/off-tumor effects. Engagement of normal endothelium could lead to endothelial activation and the release of pro-inflammatory cytokines, such as IL-6, potentially contributing to systemic inflammatory responses, increased vascular permeability, or cytokine release–associated effects.^62^ These considerations should be carefully taken into account when evaluating the therapeutic window and safety profile of B7-H3–directed CD3-engaging strategies in early-phase clinical studies.

Approximately 50% of PeCas are associated with high-risk human papillomavirus (HPV) infection, suggesting the potential relevance of HPV-targeted immunotherapies in this disease. Therapeutic HPV vaccines are designed to elicit robust CD8⁺ cytotoxic T cell responses against HPV-positive tumors by targeting the viral oncoproteins E6 and E7, which are constitutively expressed in infected cells following integration of the HPV genome.^63^ Although no clinical trials have specifically focused on therapeutic HPV vaccines in PeCa, patients with the disease are included in several ongoing basket trials enrolling individuals with HPV-associated tumors, often evaluating HPV vaccines in combination with systemic immunotherapies.^64^ Moreover, engineered T cells targeting HPV antigens, such as the viral oncoproteins E6 and E7, have demonstrated the ability to induce tumor regression in HPV-associated epithelial cancers.^65^ Ongoing early-stage studies are now extending this therapeutic approach to patients with HPV-positive PeCa.^66^

Immune checkpoint inhibitors have also been investigated in small cohorts of PeCa patients. The ongoing HERCULES trial (LACOG 0218, NCT04224740), which evaluates the combination of platinum-based chemotherapy with pembrolizumab as first-line treatment, has demonstrated an overall response rate of approximately 40%. However, this benefit is accompanied by significant toxicity, with grade 3–4 adverse events reported in 51.4% of patients. In contrast, immune checkpoint inhibitors used as monotherapy show substantially lower response rates, particularly in the first-line setting, where responses as low as 10% have been observed.^67^,^68^ These results underscore a critical constraint in contemporary immunotherapeutic strategies for PeCa, which are predominantly confined to HPV-positive cases or demonstrate limited efficacy as monotherapies. In contrast, B7-H3 was consistently and strongly expressed in all tested patient-derived PeCa samples, rendering it a more universally applicable and potentially superior target for T cell-based therapies, including bsAbs such as CC-3. This broader applicability could allow more patients, regardless of HPV status, to benefit from immunotherapy.

CC-3 incorporates a low-affinity CD3-binding domain to reduce cytokine release while preserving anti-tumor activity.^30^ Previously, CC-3 demonstrated efficacy in gastrointestinal cancers, and murine models, where it effectively suppressed lung metastases, controlled subcutaneous tumor growth, and eliminated established tumors, without inducing off-target T cell activation.^30^,^31^ BsAbs offer distinct advantages, including tumor-specific T cell-redirection with a lower incidence of adverse effects, typically limited to cytokine release syndrome. BsAbs can induce a targeted anti-tumor response while minimizing the off-target effects commonly observed with other immunotherapeutic agents.^69^ This selective mechanism may improve treatment tolerability, particularly in patients with comorbidities and/or with advanced-stage disease, by reducing therapy-related impacts on quality of life. Moreover, the favorable safety profile opens the possibility of treating high-risk patients with suspected micrometastatic disease before it becomes clinically detectable.

For clinical translation, a first-in-human prospective multicenter trial (NCT05999396) is being conducted in patients with colorectal, sarcoma, and breast cancer who have progressed after available guideline-recommended systemic therapies. Eligible patients must have measurable disease according to RECIST 1.1, an ECOG performance status ≤2, adequate organ function, and no active infection, significant central nervous system pathology, or ongoing autoimmune disease. The study applies an accelerated titration dose-escalation design beginning with single-patient cohorts starting at 50 µg CC-3, with dose increases of up to 100% between patients under the supervision of an independent safety review committee. Upon occurrence of grade ≥2 adverse events, dose-limiting toxicity, or reaching a dose level of ≥800 µg, escalation switches to a conventional 3+3 dose-escalation design. After determination of the maximum tolerated dose (MTD), an expansion cohort of 14 patients will receive CC-3 at the MTD to further assess safety and preliminary efficacy, while safety is ensured through strict eligibility criteria, predefined dose-limiting toxicity thresholds, continuous monitoring of adverse events according to CTCAE v5.0, and oversight by an independent safety review committee.^33^

## Conclusions

The results of this study, derived from patient-derived tumor and immune cells, support the therapeutic relevance of B7-H3-targeting in PeCa. Our findings demonstrate B7-H3 expression in penile cancer and show CC-3–mediated T cell activation and tumor cell killing in in vitro and ex vivo models, thereby offering an exploratory translational rationale for further investigation. Our data provide preclinical evidence to reinforce the inclusion of PeCa patients in ongoing clinical trials. A clinical basket trial (NCT05999396) has been initiated to evaluate the safety and efficacy of the CC-3 in patients with metastatic colorectal, sarcoma and breast cancer. Given the supportive in vitro and ex vivo data, inclusion of PeCa patients in this ongoing trial is now being pursued.

## Data Availability

The data generated in this study are available upon request from the corresponding author.
